# The Role of HPA Axis and Allopregnanolone on the Neurobiology of Major Depressive Disorders and PTSD

**DOI:** 10.3390/ijms22115495

**Published:** 2021-05-23

**Authors:** Felipe Borges Almeida, Graziano Pinna, Helena Maria Tannhauser Barros

**Affiliations:** 1 Graduate Program in Health Sciences, Federal University of Health Sciences of Porto Alegre (UFCSPA), Rua Sarmento Leite, 245, Porto Alegre 90050-170, RS, Brazil; felipeba@ufcspa.edu.br (F.B.A.); helenbar@ufcspa.edu.br (H.M.T.B.); 2 The Psychiatric Institute, Department of Psychiatry, College of Medicine, University of Illinois at Chicago, 1601 W. Taylor Str., Chicago, IL 60612, USA

**Keywords:** stress, hypothalamus-pituitary-adrenal axis, neurosteroids, depression, brexanolone, PTSD

## Abstract

Under stressful conditions, the hypothalamic-pituitary-adrenal (HPA) axis acts to promote transitory physiological adaptations that are often resolved after the stressful stimulus is no longer present. In addition to corticosteroids (e.g., cortisol), the neurosteroid allopregnanolone (3α,5α-tetrahydroprogesterone, 3α-hydroxy-5α-pregnan-20-one) participates in negative feedback mechanisms that restore homeostasis. Chronic, repeated exposure to stress impairs the responsivity of the HPA axis and dampens allopregnanolone levels, participating in the etiopathology of psychiatric disorders, such as major depressive disorder (MDD) and post-traumatic stress disorder (PTSD). MDD and PTSD patients present abnormalities in the HPA axis regulation, such as altered cortisol levels or failure to suppress cortisol release in the dexamethasone suppression test. Herein, we review the neurophysiological role of allopregnanolone both as a potent and positive GABAergic neuromodulator but also in its capacity of inhibiting the HPA axis. The allopregnanolone function in the mechanisms that recapitulate stress-induced pathophysiology, including MDD and PTSD, and its potential as both a treatment target and as a biomarker for these disorders is discussed.

## 1. Introduction

The adaptation to adverse conditions is a feature of utmost importance for life preservation in all organisms. Complex systems have evolved to enable a dynamic shifting of biological functions with multiple feedback mechanisms to reset the system back to homeostatic conditions. Abnormal exposure to stressors may disbalance this system and exacerbate neuroendocrine dysfunction [1]. Indeed, maladaptive responses to stress have been implicated in the development of multiple physical and neuropsychiatric disorders. Even though the precise etiology of such disorders remains largely unknown, the concept that both individual susceptibilities (determined by genetics and epigenetic factors) and stressful conditions (determined by the environment) contribute to the onset of psychiatric disorders is well accepted [2,3]. The magnitude and periodicity of stressors, in combination with an individual’s genetic construct, are also important factors that may predict what condition may arise after stress exposure. Chronic, repeated stress is believed to be an important risk factor in the development of major depressive disorder (MDD) [4], while exposure to a single, yet extremely intense traumatic event in combination with chronic stressful conditions may lead to anxiety spectrum disorders or precipitate posttraumatic stress disorder (PTSD) [5,6].

Understanding stress-related biologic alterations underlying these conditions is useful not only for a diagnostic perspective, but also to unveil targets for novel pharmacological interventions. In this review, we summarize some pathophysiological mechanisms that are involved in the hypothalamic pituitary adrenal (HPA) stress response and that are altered in MDD and PTSD. We discuss the benefits and pitfalls of promising markers regulating stress response encompassing the HPA axis. These entail the promise of improving future diagnostic accuracy of psychiatric disorders, as well as providing novel treatments. Even though only a subgroup of PTSD or depressed patients displays alterations of the HPA axis, targeting the HPA axis components might add to the treatment of PTSD or may serve as adjunct treatment in treatment-resistant subjects.

The stress response is highly complex and involves several neurobiological alterations in neurotransmitter systems, peptides, neuro-hormones, endocannabinoids, and other endogenous molecules. Here, our main focus is the intriguing role of allopregnanolone in modulating the HPA response in acute and chronic stress that may bring new diagnostic and treatment options for MDD and PTSD.

## 2. Stress, the HPA Axis and Mood Disorders

Major life events such as early or adulthood life stress are associated with increased inflammatory processes that can mediate depression and other mood disorders. Two main physiological pathways are involved in converting social-environmental adversity into broad proinflammatory transcriptional modifications that play a role in stress-induced mood disorders: the sympathetic nervous system (SNS), and the HPA axis [7].

In response to a stressor, the paraventricular nucleus (PVN) of the hypothalamus releases corticotropin releasing hormone (CRH) and arginine vasopressin (AVP). While AVP activates the locus ceruleus-norepinephrine (LC-NE) neuromodulatory system that triggers the behavioral “fight or flight” response from the SNS (mediated by epinephrine and norepinephrine), CRH acts on the pituitary gland, which in response secretes adrenocorticotropic hormone (ACTH) into the bloodstream. Once ACTH reaches the adrenal glands, it triggers the release of cortisol (in humans) or corticosterone (in rodents), which acts as anti-inflammatory hormones, and coordinates the physiological behavioral response to stress. Under normal conditions, the negative feedback of cortisol on CRH and ACTH ensures HPA homeostasis through the activation of glucocorticoid receptors (GRs) and mineralocorticoid receptors (MRs), which will act mainly by regulating gene transcription. Occupancy of MRs is generally high even in the lower levels of the circadian rhythm of glucocorticoids, and thus, the GR plays an important role in the stress response, whether this is acute or chronic [8]. This process of dynamic adaptation to reach homeostasis in the face of adverse conditions is termed allostasis, and the accumulation of such changes over time is called the allostatic load [9]. A high allostatic load, due to chronic exposure to stress, may lead to a process known as glucocorticoid insensitivity, and less negative feedback by glucocorticoids may occur when cells become less sensitive to the effects of over-secreted glucocorticoids [7]. 

Alterations in the HPA axis have been consistently reported in subjects suffering from MDD or PTSD. About 30% of depressed individuals display higher levels of cortisol [10,11], and subjects in remission present levels comparable to healthy, never-depressed controls [12]. A recent meta analysis conducted to determine cortisol level change as a biomarker for depression confirmed that cortisol measured in saliva is a predictor, but with small predictive effect on onset or relapse, and recurrence of subsequent MDD [13]. The high cortisol levels and the lack of diurnal oscillation of cortisol in mood disorders has been associated with lack of suppression to its release induced by chronic stress and inflammation. The dexamethasone (DEX) suppression test was widely studied in the 1970s and 1980s as a diagnostic tool for psychiatric disorders, including MDD, given that about 66% of depressed patients show inability to suppress cortisol release after DEX administration [14,15]. Low diagnostic sensitivity and specificity for MDD was subsequently demonstrated [16], which was later improved by the addition of CRH administration in a refined DEX/CRH test [17,18]. The DEX suppression test was also proposed as a predictor of individual treatment response to antidepressant treatment. An elevated neuroendocrine response to the combined DEX/CRH test can be detected during an acute depressive episode, but it fails to represent specificity in depression given that this response is common to other psychiatric disorders as well as several medical conditions [19]. The possibility that non-suppression of cortisol in the DEX/CRH test might constitute a biomarker for subpopulations of a given psychiatric disorder and/or might predict a higher antidepressant treatment efficacy is under discussion.

Endocrine alterations pertaining to the HPA axis are also present in PTSD, though they are qualitatively different from those found in MDD. Basal cortisol levels are often found to be reduced in PTSD patients [20]. A recent meta-analysis found lower morning salivary cortisol in PTSD patients compared to healthy subjects [21,22]. The hypoactivity of the HPA axis found in PTSD patients relates to an enhanced responsiveness and expression of GRs, which in turn facilitates the negative feedback mechanism [20]. This imbalance involving low cortisol and high GR-mediated signaling predicts a higher response to DEX. Although there are conflicting data regarding patients with PTSD and DEX-induced cortisol suppression, a majority of studies does in fact point to a higher suppression of cortisol in the DEX suppression test than in trauma-exposed healthy subjects [23]. Additionally, the HPA axis response to stressors seems to be altered in individuals suffering from PTSD. In women, an impaired cortisol response to a stress test was found in PTSD but not in MDD patients [24], which was also replicated in men with PTSD [25].

The HPA axis response to stress can also be regulated by GABAergic signaling, mainly through the activation of GABA type A receptors (GABA_A_Rs) [26]. GABA_A_Rs are ligand-gated ionotropic receptors that promote the influx of Cl^−^ and subsequent hyperpolarization of postsynaptic neurons when activated. In light of the heteropentameric structure, a large number of receptor subtypes can be composed with different synaptic GABA_A_Rs localization that participate both in *phasic* inhibition (fast action mediated by synaptic receptors), as well as *tonic* inhibition (slow and persistent form of inhibition mediated by extrasynaptic receptors) (reviewed in [27]). Brain regions involved in the regulation of the HPA axis make GABAergic connections with the PVN and its surrounding area, and thus CRH neurons receive many GABAergic inputs. These originate from the subparaventricular zone, the anterior hypothalamic area, dorsomedial hypothalamic nucleus, the medial preoptic area, lateral hypothalamic area, and some subnuclei in the bed nucleus of the stria terminalis [28]. Studies in animals have supported this hypothesis, since heterozygous γ2^+/−^ mice show overactivity of the HPA axis [29]. Additionally, the infusion of the GABA_A_R antagonist, bicuculline, into the PVN accentuates the increase in corticosterone caused by stress, while the infusion of the GABAmimetic, muscimol, in the same region, dampens the corticosterone increase after stress [28]. Thus, endogenous GABAergic modulators, particularly those with action at GABA_A_Rs, have been proposed as prominent treatment strategies for the treatment of MDD and PTSD. One such category of endogenous GABA_A_R neuromodulators constitutes the neurosteroids, allopregnanolone and its equipotent isomer, pregnanolone.

Several other possible mechanisms of action of allopregnanolone deserve to be acknowledged due to their importance for the treatment of mood disorders. Beyond the GABAergic system, allopregnanolone also exerts modulatory effects on calcium channels, autophagy mechanisms, expression of nuclear receptors, hippocampal neurogenesis, among other mechanisms [30]. In this review we focus on the GABAergic mechanism of action of allopregnanolone, however, it is relevant to point out that other important molecular pathways may contribute to its antidepressant effect. Its involvement in the glutamatergic neurotransmission and in BDNF signaling was reviewed in previous outstanding contributions to this topic [31,32].

## 3. Allopregnanolone Biosynthesis Implication in Stress Response

**Figure 1:**
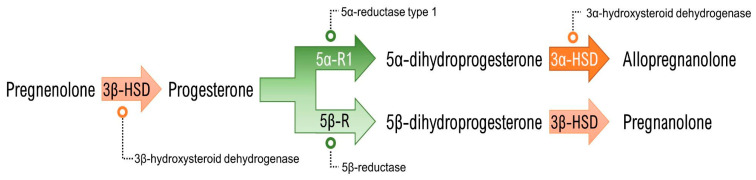
Allopregnanolone (and its isomer pregnanolone) synthesis pathway from pregnenolone.

Allopregnanolone is defined as a neuroactive steroid and a neurosteroid [33], in that: (a) it possesses a steroidal structure and that (b) its synthesis takes place, at least partially, in the central nervous system, which defines it as a neurosteroid. It may also be synthesized in the adrenal glands, ovaries and testicles and may act as a neuroactive steroid after reaching and acting in the brain. Regardless of the site of synthesis, the process of neurosteroidogenesis begins with the trafficking of cholesterol to the outer mitochondrial membrane and its subsequent internalization to the inner mitochondrial membrane by the action of the steroidogenic acute regulatory (StAR) protein and the 18 kDa translocator protein (TSPO). Once in the inner mitochondrial membrane, the crucial step of cholesterol side chain cleavage takes place by action of the CYP11A1 enzyme, and the neurosteroid precursor pregnenolone is produced. By the action of the 3β-hydroxysteroid dehydrogenase (3β-HSD) enzyme, pregnenolone can be converted into progesterone. Beyond being a precursor of allopregnanolone and many other pregnan steroidal molecules, progesterone is also a sex hormone with a large role on the regulation of physiological and behavioral effects. Relevant for the allopregnanolone synthesis pathway (Figure 1) is the action of the 5α-reductase enzyme, which exists in two main isoforms (type 1 and type 2, abbreviated 5α-R1 and 5α-R2, respectively). 5α-R1 is the most abundant isoform in the brain and is the principal agent in converting progesterone to 5α-dihydroprogesterone (5α-DHP), which is the final precursor to allopregnanolone. 5α-DHP is converted to allopregnanolone by action of the 3α-hydroxysteroid dehydrogenase (3α-HSD), a cytosolic enzyme found in the brain and in steroidogenic tissues as well. It is worth highlighting that pregnanolone—the equipotent isomer of allopregnanolone—is also synthesized from progesterone through the action of the 5β-reductase (5β-R) enzyme, which originates 5β-dihydroprogesterone (5β-DHP). 5β-DHP is also transformed by 3α-HSD into pregnanolone [34,35].

**Figure 2:**
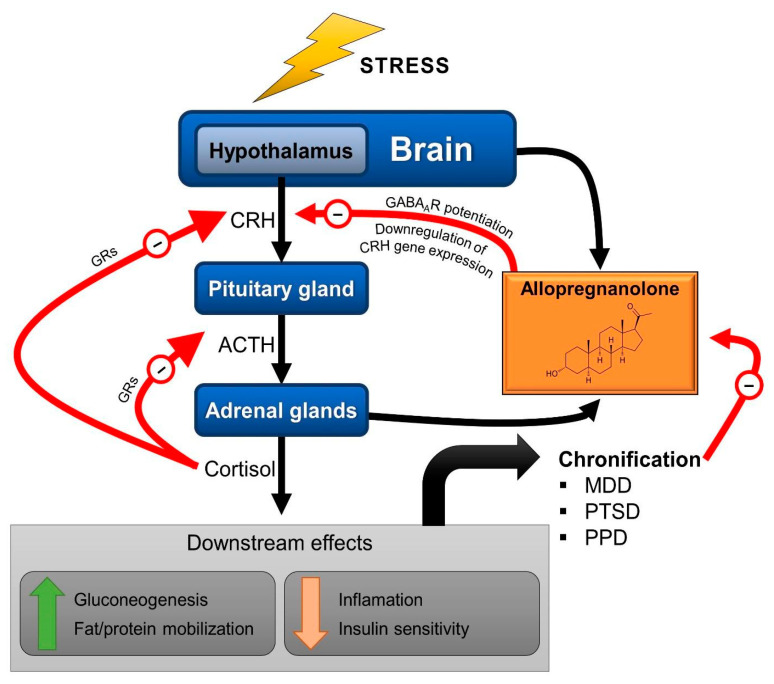
Schematic representation of the HPA axis with the neurosteroid allopregnanolone as a modulator of the stress response. Through positive allosteric modulation of the GABA_A_ receptors (GABA_A_Rs) present in corticotropin-releasing hormone (CRH) neurons, allopregnanolone participates in the negative feedback that eventually terminates the acute stress response. ACTH: adrenocorticotropic hormone; GRs: glucocorticoid receptors; MDD; major depressive disorder; PTSD: post-traumatic stress disorder; PPD: postpartum depression. Red arrows (−) indicate negative feedback.

Allopregnanolone and pregnanolone are capable of modulating neuronal activity, specifically potentiating GABAergic neurotransmission due to their potent, positive and allosteric modulation of the action of GABA at GABA_A_Rs. Importantly, pretreatment with allopregnanolone attenuates the endocrine response to stress [36,37], probably owing the GABA-mediated inhibition of CRH neurons. This appears to occur following tonic activation of extrasynaptic GABA_A_Rs containing the δ subunit, which makes them particularly sensitive to neurosteroids [38]. Additionally, allopregnanolone acts through genomic pathways, downregulating the gene expression of CRH [36] and of AVP [37] in the hypothalamus of rats. These findings place allopregnanolone as an important neuromodulator involved in the regulation of stress response and as part of the allostatic mechanisms exerting a negative feedback on the HPA axis (Figure 2).

Indeed, as first demonstrated by Purdy and colleagues, exposure to acute stress quickly elevates allopregnanolone levels in both plasma and brain [39]. This stress-induced surge in brain and periphery allopregnanolone levels was further replicated using other stressors, such as CO_2_ inhalation [40] or foot shock [41,42]. Even though allopregnanolone surges in both brain and plasma in response to acute stress, intriguingly, each event is characterized by distinct kinetics. Purdy and colleagues also demonstrated in their seminal findings [39] that, although a majority of the allopregnanolone increase in the brain originates from peripheral sources, even in adrenalectomized rats there is a significant cortical increase that demonstrates the importance of its de novo synthesis in the brain. Furthermore, allopregnanolone increases more quickly in the brain compared to plasma [39]. This acute stress-induced increase in allopregnanolone is probably part of a wider mechanism that aims to promote homeostasis and that regulates HPA axis activation due to their action on GABAergic neurotransmission. Studies in handling-habituated rats (i.e., rats accustomed to the manipulation and process involved immediately before sacrifice) as ‘unstressed’ controls have found a significant reduction in [^3^H]GABA binding at GABA_A_Rs after a single foot shock, which indicates a rapid reduction in GABAergic neurotransmission following acute stress [43]. Allopregnanolone then surges in the brain and acts to restore homeostasis by positively potentiating GABA_A_Rs, restoring GABAergic neurotransmission.

## 4. Chronic Stress and Its Role in MDD, PTSD and Allopregnanolone Levels

When an individual is subjected to one or more stressors for extended periods of time in a significant frequency during a given time period of significant length, long-term effects on physiological parameters begin to appear. Such effects are mostly demonstrated by increased levels of stress hormones, such as cortisol. These modifications define the allostatic load, which can manifest in the incapacity of adaptation to a stressor or the inability to properly terminate the stress response [9]. The repeated activation of the HPA axis leads to neuroendocrine adaptations that impair the secretion of glucocorticoids and throw off the balance that these stress mediators exert over other neural systems involved in stress response.

Importantly, chronic stress has been implicated in the development of MDD, particularly in the context of precipitating depressive episodes [44,45]. Chronic stress is also believed to play a role in the exacerbation of PTSD symptomatology, with MDD frequently being comorbid with PTSD. This finding is in kind with the observation that an impaired HPA axis response is frequently observed in MDD and PTSD (as discussed in Section 3, above), strengthening the link between the chronification of stress and the appearance of depressive manifestations. In addition to the high circulating cortisol levels and abnormal response in the DEX or DEX/CRH suppression test, depressed patients also present alterations in peripheral allopregnanolone levels, which have been found to be decreased in serum [46], plasma [47,48] and cerebrospinal fluid [49]. The mechanisms orchestrating this complex pathophysiological response that contrasts acute and chronic levels of allopregnanolone are not fully understood. Some studies show that increased allopregnanolone levels due to persistent or repetitive stress may induce tolerance probably by altering the sensitivity of GABA_A_Rs [50,51], particularly through changes in subunit composition. Chronic administration of allopregnanolone decreased the mRNA levels of β2, β3, α2 and α3 subunits in the mammalian cortex, without changing γ2 expression [52]. It is hypothesized that chronic stress, through yet unknown mechanisms, may attenuate the upregulation of steroidogenic enzymes in order to reduce allopregnanolone levels and regain GABA_A_R sensitivity. However, experimental data are still needed to elucidate whether this is in fact the mechanism behind chronic stress-induced allopregnanolone downregulation (as reviewed in [53]).

Interestingly, sex dimorphism appears to be an important factor for the alterations in allopregnanolone levels in PTSD patients. Women with PTSD show decreased cerebrospinal fluid levels of allopregnanolone with unchanged levels of progesterone and 5α-DHP, which indicated an impairment in the 3α-HSD enzyme function [54]. Male PTSD patients also present lower allopregnanolone levels in the cerebrospinal fluid that correlated inversely with depressive symptoms, but the results point to a deficit in the 5α-R1 enzyme function or expression [55]. The deficit in 3α-HSD function in women with PTSD was also demonstrated using much less invasive methods than the lumbar puncture. Allopregnanolone measured in the plasma of female patients with PTSD confirmed a conversion deficit of progesterone to allopregnanolone compared to trauma-exposed women without PTSD [56,57]. These findings, together with the increasing ability to consistently and more reliably measure neurosteroids, potentially even in easy-access specimens, including saliva, lends an interesting perspective on their use as biomarkers of MDD and PTSD [35].

Indeed, many of the protocols designed for animal studies that are frequently used to model MDD involve the application of chronic stressors that are accompanied by similar alterations in the HPA axis [4]. In the same direction, chronic administration of corticosterone is used as a rodent model of depression that is characterized by high immobility in the forced swim test and by impaired hippocampal neurogenesis. The latter can be mediated by reelin, a neurotrophin characteristic of GABAergic interneurons [58]. Additionally, low allopregnanolone levels are also observed in the brain of rodents submitted to animal models of depression based on chronic stress protocols, such as the social isolation [59] and chronic unpredictable stress (reviewed in [32]). Another interesting finding is that, in socially isolated male rats, the acute stress-induced increase in allopregnanolone is even greater than in group-housed animals, even though their basal levels are lower, indicating a dysregulation in allopregnanolone biosynthesis caused by chronic stress [60].

Less clear is the role of chronic stress in the pathophysiology of PTSD. The efforts to model PTSD in animals have largely focused on the induction of a traumatic event of sufficient magnitude to induce behavioral alterations reproducing the symptoms of the disorder in humans. This paradigm is in line with the understanding that acute/unique—rather than chronic/repeated—stress is in the center of the pathophysiology of PTSD. In humans, PTSD is a protracted and persisting disorder with post-trauma symptoms that fail to be extinguished. Thus, the best models should be those that are able to distinguish alterations in fear extinction and fear extinction retention after trauma exposure [57,61]. Some models that feature longer exposures to stress (e.g., social defeat stress) or even chronic stress exposure (e.g., social isolation) have also been proposed to be relevant for the study of the vulnerability to PTSD, presenting several of the neurobiological characteristics of the disorder [62]. Though less directly translatable to the clinical manifestation of PTSD, these models may be useful to investigate several facets of this highly heterogeneous disorder.

## 5. Allopregnanolone-Based Therapeutics as a Treatment Option for MDD and PTSD

The regulatory capacity that allopregnanolone exerts on the HPA axis indicates a potential target for the normalization of the neuroendocrine alterations observed following protracted stressful conditions, potentially contributing to the alleviation of symptoms and perhaps leading to remission of MDD and PTSD. The potentiation of GABAergic neurotransmission on CRH neurons via exogenous allopregnanolone administration could therefore be a possible strategy for the normalization of the HPA axis responsiveness in PVN neurons, at the same time that this neuromodulator improves behavioral alterations by facilitating corticolimbic GABAergic signaling.

Such a therapeutic approach has also shown clinical efficacy with the intravenous administration of brexanolone (a pharmaceutical preparation of allopregnanolone marketed as Zulresso™) for the treatment of postpartum depression (PPD) [63,64]. Being the first neurosteroid to receive F.D.A. approval for the treatment of depressive disorders [65], brexanolone may very well be the starting point of a whole novel field of neurosteroid-based pharmacotherapies for the treatment of psychiatric disorders. Even though PPD is a specific subtype of depressive disorders that affects only a particular population of subjects (i.e., women in the perinatal period), alterations in the HPA axis have been implicated in its pathophysiology as well [66]. Perhaps due to the dynamic and intense nature of the changes in endocrine regulation during pregnancy [67], there are conflicting findings regarding the specific nature of HPA axis alterations in PPD. According to a recent systematic review, the best evidence points to an attenuated response to either physiological or non-physiological stimuli in women suffering from PPD [68]. Both the clinical success of brexanolone for the treatment of PPD and the data from animal studies that show allopregnanolone-induced positive behavioral responses associated with the normalization of the HPA axis function [66] provide an encouraging setting for its therapeutic application in related psychiatric disorders, particularly MDD. The HPA axis and allopregnanolone alterations found in PTSD (albeit distinct from those found in MDD) suggest a promising prospect for allopregnanolone-based treatments for this condition as well, but more research is needed to clarify this topic.

Clinical data on antidepressant effects of allopregnanolone are mostly restricted to PPD, however, several clinical trials are ongoing to study the efficacy of similar molecules in the treatment of MDD. One such example is the allopregnanolone analog SAGE-217, which, despite its recent clinical trial failure due to the absence of detectable blood levels in 10% of patients, has shown positive effects in patients that presented significant drug levels. In these patients, a statistically significant decrease in symptoms measured by the Hamilton Depression Rating Scale was achieved in days 3, 8, 12, and 15 after treatment with SAGE-217 [69]. Additionally, recent results with ganaxolone, an allopregnanolone analogue, treatment-resistant depression of post-menopausal women, point out that it may be a useful adjunctive in patients with depression and insomnia [70]. 

In animal models, allopregnanolone antidepressant-like effects after systemic or intracerebroventricular administration has been largely demonstrated in the forced swim test, a well-validated model for assessing antidepressant action [71,72,73,74,75,76,77]. Furthermore, upregulation of brain neurosteroid levels by antidepressant drugs such as the selective serotonin reuptake inhibitors (SSRIs) fluoxetine and norfluoxetine in socially isolated mice has been shown to mediate, at least partially, the amelioration of behaviors with relevance to PTSD, such as aggressive-like behaviors [78,79,80]. In fact, because of the significant magnitude of this effect, and because the upregulation of neurosteroidogenesis occurs independently of any downregulation of serotonin reuptake, these drugs were considered to also act as selective brain steroidogenic stimulants (SBSSs), underscoring a whole novel antidepressant mechanism for well-established antidepressants [81]. Furthermore, the allopregnanolone analog ganaloxone, when administered in socially isolated mice, has also exerted antiaggressive and anxiolytic-like behaviors in non-sedative doses [82], even when mice were socially isolated from the early adolescent period [83]. Indeed, another allopregnanolone analog, SGE-516, also showed promising results in mouse models of PPD based on absent or reduced expression of the δ GABA_A_R subunit or lack of the K^+^/Cl^−^ co-transporter (KCC2). Both δ-containing GABA_A_Rs and KCC2 are important for effective GABAergic inhibition, specifically in the regulation of CRH neurons. SGE-516 was able to prevent the stress-induced increase in corticosterone and the dephosphorylation and downregulation of KCC2, while decreasing depression-like behaviors observed during the postpartum period [84].

Overall, the neuromodulatory role of allopregnanolone and its analog molecules on GABAergic neurotransmission, particularly acting on the regulation of the HPA axis, may represent a main mechanism underlying the treatment of MDD and PTSD. Stimulation of allopregnanolone biosynthesis by the development of novel, highly selective neurosteroidogenic drugs may become a reality in the near future and provide a much-needed diversification in the pharmacological treatment of these debilitating disorders [30,33,69].

## 6. Conclusions

Allopregnanolone plays a regulatory role on the neuroendocrine alterations resulting from the stress response, particularly in conjunction with the feedback mechanisms regulating the HPA axis, mediated by GABA_A_Rs. Both preclinical and clinical studies have observed allopregnanolone’s rapid increase in acute stress and persistently diminished levels in chronic stress. Future studies are required to determine the role of GABAergic dysfunction, altered neurosteroid signaling in stress, and HPA axis dysregulation in MDD and PTSD as well as the usefulness of HPA biomarkers associated with neurosteroid biosynthesis in the diagnosis and treatment of these disorders.
